# The Diseases of Children

**Published:** 1900-03

**Authors:** 


**The Diseases of Children.** By GEORGE ELDER, M.D., and J. S. FOWLER, M.B. Pp. xiii., 391. London: Charles Griffin and Company, Limited. 1899.

The list of clinical handbooks published by Messrs. Griffin has received a useful addition in the volume before us. Though we do not as a rule like books which at best profess to be only half-measures, we confess to a decided liking for this one; for with small type and closely-set lines, much more is contained in it than in many similar books professing more. The few illustrations which are inserted are good and typical examples of the conditions or diseases they represent. We think students will find the work a useful one; but it requires some knowledge of general medicine to fully appreciate much that it contains, as the authors have not quite written down to a student's level. Possibly they intended it for senior students and practitioners, though for the latter some parts, such as treatment, are not sufficiently full, a fault only too common in works on children's diseases.

Medical work in this department has suffered a distinct loss by the recent premature death of Dr. Elder, who at the early age of thirty has been taken from us by an attack of influenza.

